# Novel strategy with the automatic non-coplanar volumetric-modulated arc therapy for angiosarcoma of the scalp

**DOI:** 10.1186/s13014-020-01614-3

**Published:** 2020-07-17

**Authors:** Shoki Inui, Yoshihiro Ueda, Shingo Ohira, Haruhi Tsuru, Masaru Isono, Masayoshi Miyazaki, Masahiko Koizumi, Teruki Teshima

**Affiliations:** 1 Department of Radiation Oncology, Osaka International Cancer Institute, 3-1-69 Otemae, Chuou-ku, Osaka, 537-8567 Japan; 2 Department of Medical Physics and Engineering, Osaka University Graduate School of Medicine, Suita, Japan

**Keywords:** Total scalp irradiation, HyperArc, VMAT, Flattening filter free, Angiosarcoma of the scalp

## Abstract

**Background:**

Total scalp irradiation presents technical and dosimetric challenges. While reports suggest that HyperArc, a new stereotactic radiosurgery planning technique applied to non-coplanar volumetric-modulated arc therapy (VMAT) technique, is associated with high conformity and rapid dose fall-off, the performance of HyperArc for total scalp irradiation has not been explored. The current study aimed to compare the dosimetric performance of HyperArc plans with those of non-coplanar VMAT plans in angiosarcoma of the scalp.

**Methods:**

Ten patients with angiosarcoma of the scalp were included in this study. The performance of three different plans administered using TrueBeam Edge were compared: non-coplanar VMAT using flattening filter (FF) beams (VMAT-FF), HyperArc using FF beams (HyperArc-FF), and HyperArc using flattening filter free (FFF) beams (HyperArc-FFF). The dose distribution, dosimetric parameters, and dosimetric accuracy for each of these plans were evaluated.

**Results:**

The three plans showed no statistically significant differences in target volume coverage, conformity, and homogeneity. The HyperArc-FF and HyperArc-FFF plans provided significantly lower mean brain doses (12.63 ± 3.31 Gy and 12.71 ± 3.40 Gy) than did the VMAT-FF plans (17.11 ± 5.25 Gy). There were almost no differences in sparing the organs at risk between the HyperArc-FF and HyperArc-FFF plans. The HyperArc-FF and HyperArc-FFF plans provided a shorter beam-on time than did the VMAT-FF plan. The 3%/2 mm gamma test pass rates were above 95% for all three plans.

**Conclusions:**

Our results suggest that the HyperArc plan can be potentially used for radiation therapy of target regions with large and complicated shapes, such as the scalp, and that there are no advantages of using FFF beams.

## Background

Angiosarcoma of the scalp is a skin malignancy, which often shows local recurrence [1, 2]. Radiation therapy is one of the most important treatment approaches for angiosarcoma of the scalp, and previous studies have reported about local control of this malignancy via total scalp irradiation (TSI) [3–5]. TSI has historically been associated with technical and dosimetric challenges because of the complicated shape of the target region and the close proximity of the scalp to the organs at risk (OARs), such as the brain and eyes.

To solve this complexity, various techniques for TSI with linear accelerators have been designed [6–13]. Electron beams have traditionally been chosen because of its high surface doses and the scattering of electrons at oblique surfaces. Combinations of electron and photon beams show higher dose uniformity than do electron beams alone [6, 7]. However, the dose distribution at the junction of the radiation fields is inhomogeneous. To overcome this problem, some approaches such as intensity-modulated radiation therapy (IMRT), volume-modulated arc therapy (VMAT), and helical tomotherapy (HT) have been investigated by several researchers. Ostheimer et al. indicated that coplanar VMAT plans were slightly superior to non-coplanar IMRT plans in coverage, homogeneity, and OAR protection [8]. Hu et al. showed that non-coplanar VMAT plans were associated with lower brain dose than are coplanar VMAT plans [9]. Song et al. reported that HT plans showed better coverage and homogeneity and longer treatment time than coplanar VMAT plans, and that the normal brain tissue receives lower dose with the former than with the latter plans [10]. These reports suggest for the patients with angiosarcoma of the scalp, it is important to choose the most effective technique by considering TSI target coverage, OAR sparing, and treatment time.

Recently, HyperArc (Varian Medical Systems, Palo Alto, CA) has been attracting attention as a new stereotactic radiosurgery (SRS) planning technique applied to non-coplanar VMAT technique with a single isocenter. The main characteristics of HyperArc are to make treatment planning as automated as possible, to alert the planner for a risk of the collision between machine-machine or machine-patient, and to reduce doses to the OARs while increasing the conformity of the target volume. Ohira et al. pointed out that HyperArc plans provided significantly higher conformity and rapid dose fall-off than did VMAT plans for single and multiple brain metastases [14]. However, none of the previous studies have explored the performance of HyperArc plans in patients other than those with SRS for single and multiple brain metastases. It is also important to understand the potential of HyperArc plans in reducing the dose received by normal brain tissue while maintaining the conformity of target regions with large and complicated shapes, such as the scalp.

The aim of this study was to compare the dosimetric performance of HyperArc plans with that of non-coplanar VMAT plans in angiosarcoma of the scalp. In addition, we focused on the use of flattening filter free (FFF) beams to spare the dose received by normal brain tissue in HyperArc plans. Therefore, we also compared the dosimetric parameters of the HyperArc plans using flattening filter (FF) beams with that using FFF beams. This study will contribute to expand the possibility of applying non-coplanar VMAT techniques using HyperArc.

## Methods

### Patients, simulation, and contouring

This study was approved by our ethics committee, and written informed consent was obtained from all patients. Between April 2017 and April 2020, ten patients with angiosarcoma of the scalp were treated with non-coplanar VMAT at our institute. All patients were chosen for analysis in this study. These patients were aged 56–86 years (median: 76 years) and immobilized with thermoplastic head masks in the supine position. CT simulations (Revolution HD, GE Medical Systems, Milwaukee, WI) were performed with a 2.0-mm slice thickness and 500-mm field of view with dimensions of 512 × 512 pixels. All the CT images were transferred to a treatment planning system (TPS) (Eclipse version 15.5; Varian Medical Systems, Palo Alto, CA).

The target volume and OARs such as the brain, brain stem, optic nerve, chiasm, and lens, were delineated by experienced radiation oncologists in our institute. Contouring of clinical target volume (CTV) included the entire scalp bordered by the face anteriorly and the neck to the sides and posteriorly. The planning target volume (PTV) was derived from CTV plus a symmetrical 5-mm margin. Mean PTV size and standard deviation were 518.2 ± 116.6 cm^3^ (range: 362.8–678.6 cm^3^). To achieve high target coverage, this study employed the use of a 1-cm virtual bolus which was added as structure in an Eclipse and applied to the surface of the skin around the PTV. This bolus was used for the optimizations and subsequent final dose calculations.

### Treatment planning

All treatment plans were performed using two photon beams of a linear accelerator, (TrueBeam Edge, Varian Medical Systems) equipped with high definition multileaf collimators (MLCs), the widths of which were 2.5 mm for the first 32 leaves from the central point and 5 mm for the rest. For the purpose of comparison, different treatment plans were designed for three techniques, including non-coplanar VMAT with FF beam (VMAT-FF), HyperArc with FF beam (HyperArc-FF), and HyperArc with FFF beam (HyperArc-FFF), in all patients. The VMAT-FF is a clinical plan and the others are not. All plans were optimized with a photon optimizer (PO) ver.15.0 and calculated with the analytical anisotropic algorithm (AAA) for dose calculation with inhomogeneity corrections on the 2 mm grid size. Prescribed dose, which was determined as the mean dose for PTV, was 35 × 2 Gy (70 Gy).

### VMAT plan

All VMAT-FF plans were generated using a 6 MV photon beam at a maximum dose rate of 600 MU per minute with non-coplanar arc fields. The number of isocenters, beam angle, field size and collimator angle were manually selected depending on the size and location of the target. Beam parameters, such as couch angle, collimator angle, and arc length, for each arc in VMAT-FF are summarized in Table 1. In the optimization process, the jaw tracking function was used. Moreover, normal tissue objective (NTO) and objectives for PTV were maintained at constant values to avoid bias. The optimization goals and constraints in the VMAT-FF plan are summarized in Table 2.

**Table 1:** Beam parameters for VMAT and HyperArc planning

Patient #	Beam parameter	Arc field number							
		1	2	3	4	5	6	7	8
VMAT									
1	Couch	315	315	0	30	30	90	90	
	Collimator	345	15	15	345	15	15	345	
	Arc length	180	180	360	180	180	180	180	
2	Couch	0	0	45	45	90	90		
	Collimator	340	20	345	15	350	10		
	Arc length	360	360	180	180	180	180		
3	Couch	315	315	0	45	45	90	90	
	Collimator	340	20	20	345	15	0	0	
	Arc length	180	180	360	180	180	180	180	
4	Couch	315	0	0	45	90	90		
	Collimator	80	355	5	100	350	10		
	Arc length	180	360	360	180	180	180		
5	Couch	270	315	315	0	0			
	Collimator	90	315	30	345	15			
	Arc length	180	90	90	205	205			
6	Couch	315	315	0	0	45	45	90	90
	Collimator	340	20	340	20	345	20	0	0
	Arc length	180	180	360	360	180	180	180	180
7	Couch	315	315	0	0	45	45	90	90
	Collimator	355	5	355	5	355	5	355	5
	Arc length	200	200	360	360	200	200	200	200
8	Couch	0	0	45	45	90			
	Collimator	345	15	0	30	90			
	Arc length	200	200	90	90	180			
9	Couch	315	315	0	0	45	45	90	90
	Collimator	340	20	340	20	340	20	0	0
	Arc length	180	180	360	360	180	180	180	180
10	Couch	315	315	0	0	45	45	90	90
	Collimator	345	15	340	20	345	15	0	0
	Arc length	180	180	360	360	180	180	180	180
HyperArc									
All	Couch	315	0	45	90				
	Collimator	345	5	15	45				
	Arc length	180	360	180	180

**Table 2:** Optimization goals and constrains used for VMAT and HyperArc planning

Structure	Objective	Priority	
		VMAT	HyperArc
NTO		75	
SRS NTO			100
PTV	D_99%_ > 63 Gy	100	250
	D_mean_ = 70 Gy	100	250
	D_max_ < 80 Gy	100	250
Brain	as low as possible	80	150
Brain stem	D_max_ < 54 Gy	50	100
Chiasm	D_max_ < 50 Gy	50	100
Optic nerve	D_max_ < 50 Gy	50	100
Lens	D_max_ < 6 Gy	150	150

### HyperArc plan

The HyperArc-FF and HyperArc-FFF plans were generated using a 6 MV photon beam at a maximum dose rates of 600 and 1400 MU per minute, respectively. The isocenter and field size were automatically determined based on the target structure in the HyperArc planning. In addition, four arc fields, three of which were non-coplanar, were automatically arranged as follow: one full coplanar arc without couch rotation and three half non-coplanar arcs with couch rotations of 315°, 45°, and 90°. The HyperArc plan used collimator angles of 5°, 345°, 15°, and 45° in the beam with couch rotations of 0°, 315°, 45°, and 90°, respectively (Table 1). In the optimization process, the jaw tracking function was used. Moreover, the SRS NTO was used, which was designed to generate treatment plans that featured steep dose decay in space from target-specific dose levels to low asymptotic dose levels. The optimization goals and constraints in the HyperArc plan are summarized in Table 2.

### Dosimetric comparisons

The treatment plans were evaluated according to the standard dose volume histograms (DVH) for VMAT-FF, HyperArc-FF, and HyperArc-FFF. For the evaluation of target dose, the homogeneity index (HI) was defined as follows: HI = (D_2%_ - D_98%_)/D_50%_, where D_50%_ is the median absorbed dose and D_2%_ and D_98%_ represent the doses received by 2 and 98% of the PTV. The conformity index (CI) was defined as follows: CI = (TV_PV_ × TV_PV_)/(TV × PV), where TV_PV_, TV, and PV represent the volume of the target covered by the prescription dose, target volume, and prescription isodose volume, respectively. For normal brain tissues excluding the PTV, the volumes that received a specific dose in a range of 10 to 60 Gy (V_10Gy_ − V_60Gy_) and the mean dose were compared. In addition, the doses receiving 0.1 cc of the volume for surrounding critical organs, such as the brain stem, chiasm, optic nerve, and lens, were evaluated. Total MUs and beam-on time (BOT) were compared for the three plans.

To evaluate the complexity of the MLC patterns, the modulation complexity score for VMAT (MCSV) for each plan was calculated using our in-house software (MATLAB R2016a; MathWorks, Natick, MA), and the overall MCSV was defined as the mean of the MCSV for each treatment beam. The MCSV was calculated based on the leaf sequence variability (LSV) parameter and aperture area variability (AAV) as described by Masi et al. [15]:

$$ MCSV={\sum}_{i=1}^{I-1}\left[\frac{\left(\mathrm{AA}{\mathrm{V}}_{cpi}+\mathrm{AA}{\mathrm{V}}_{cpi+1}\right)}{2}\times \frac{\left(\mathrm{LS}{\mathrm{V}}_{cpi}+\mathrm{LS}{\mathrm{V}}_{cpi+1}\right)}{2}\times \frac{M{U}_{cpi+1}}{M{U}_{arc}}\right] $$

where MU_*cpi, i + 1*_ indicates the number of MUs delivered between 2 successive control points (namely, CP_*i*_ and CP_*(i + 1)*_). The value of the MCSV decreases with an increase in modulation complexity.

### Dosimetric verification

In all treatment plans, dosimetric verification was performed by using the electronic portal imaging device (EPID, aS1200 flat panel detector, Varian Medical Systems) mounted on the linear accelerator. The square pixels of the EPID had a side length of 0.34 mm, which yielded a total area of approximately 40 × 40 cm^2^ (1190 × 1190 pixels). All EPID images were obtained in the integrated acquisition mode without any obstructions at the source-to-imager distance of 150 cm and 170 cm for FF and FFF beams, respectively. The acquired images were automatically retrieved to a commercial software (PerFRACTION version 2.0.4, Sun Nuclear Corporation, Melbourne, FL) and compared against the baseline images, which were generated from the DICOM files of treatment plan from the TPS. The quantitative evaluation of the dosimetric accuracy was performed using gamma method. Analysis criteria of 3%/2 mm and 2%/2 mm in gamma was used above a 10% maximum signal threshold.

### Statistical analysis

The paired Wilcoxon signed-rank test was performed on the data not following normal distribution (SPSS, version 24; IBM, Armonk, NY) for the statistical measurement of the differences between the following: VMAT-FF vs. HyperArc-FF, VMAT-FF vs. HyperArc-FFF, and HyperArc-FF vs. HyperArc-FFF. A *p* value below 0.05 was considered to indicate statistical significance.

## Results

**Fig. 1:**
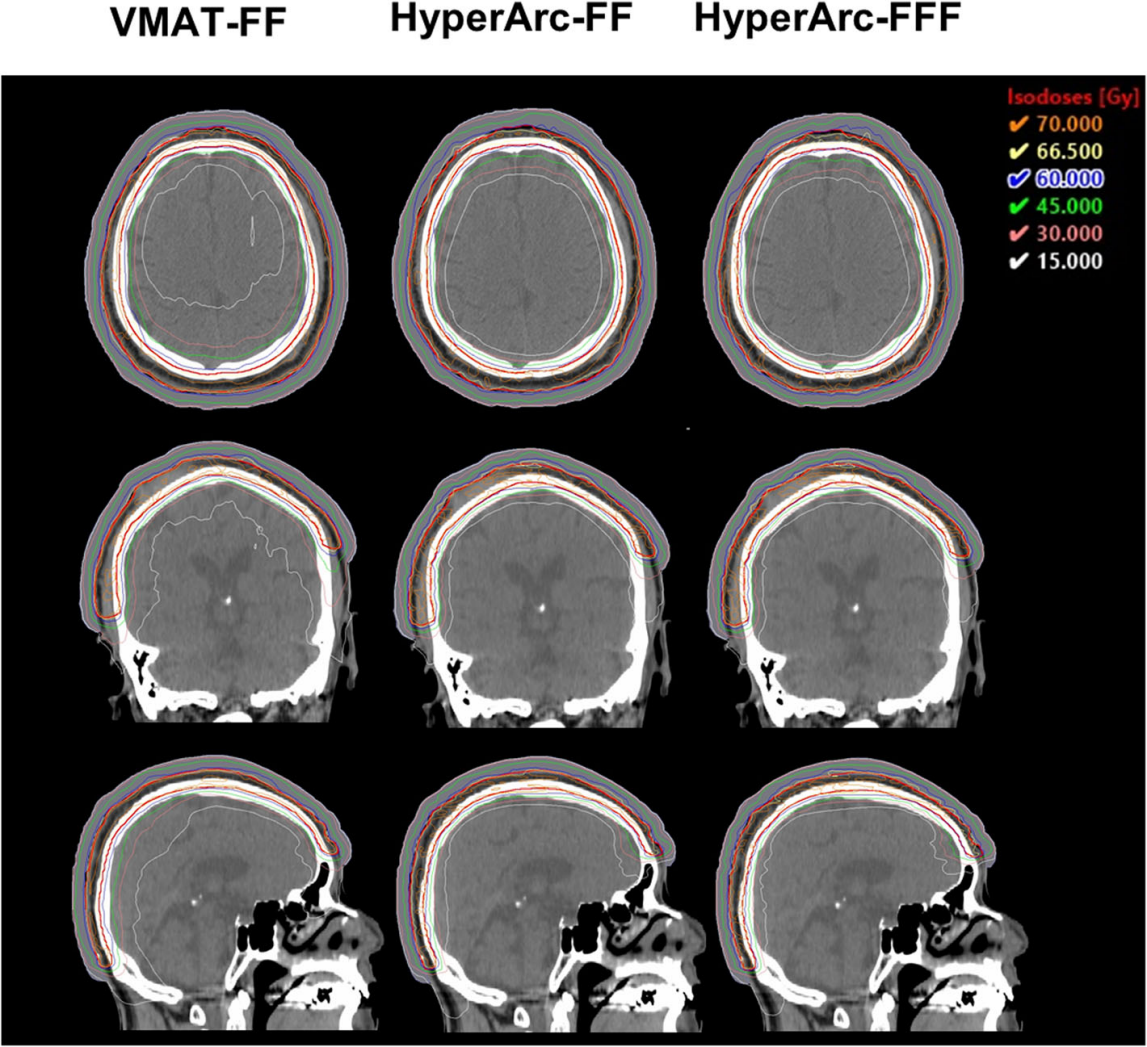
Axial, coronal, and sagittal images of the isodose distribution of the VMAT-FF, HyperArc-FF, and HyperArc-FFF plans in one patient (red; PTV, dark blue; virtual bolus)

**Fig. 2:**
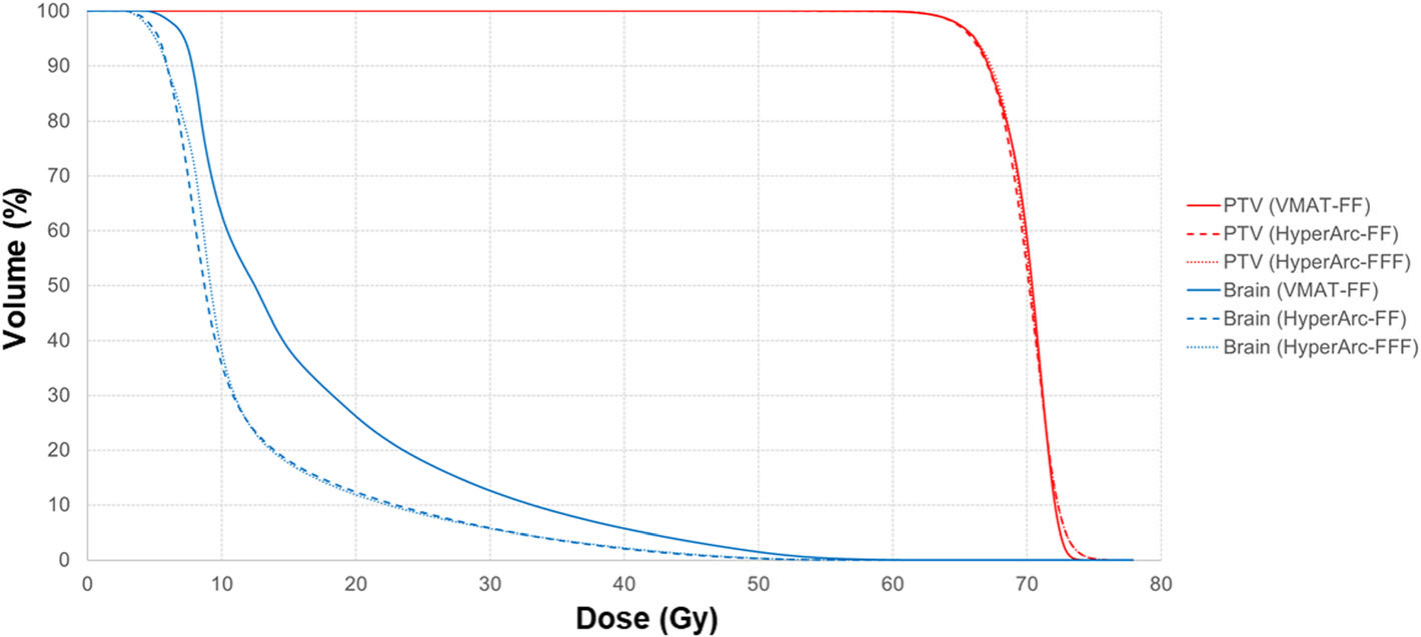
Dose-volume histogram comparison for the PTV and brain with different planning techniques in one patient

The isodose distributions among the three groups of treatment plans created with different planning techniques for one patient in axial, coronal, and sagittal views are shown in Fig. 1. The percentage of the brain tissue receiving 15 and 30 Gy for the HyperArc plans were lower than those for the VMAT plans. Figure 2 shows the DVH for the PTV and the brain in one patient. In this case, reduction in the doses received by the brain tissue while maintaining target coverage were higher with the HyperArc-FF and HyperArc-FFF plans than with the VMAT-FF plan.

**Fig. 3:**
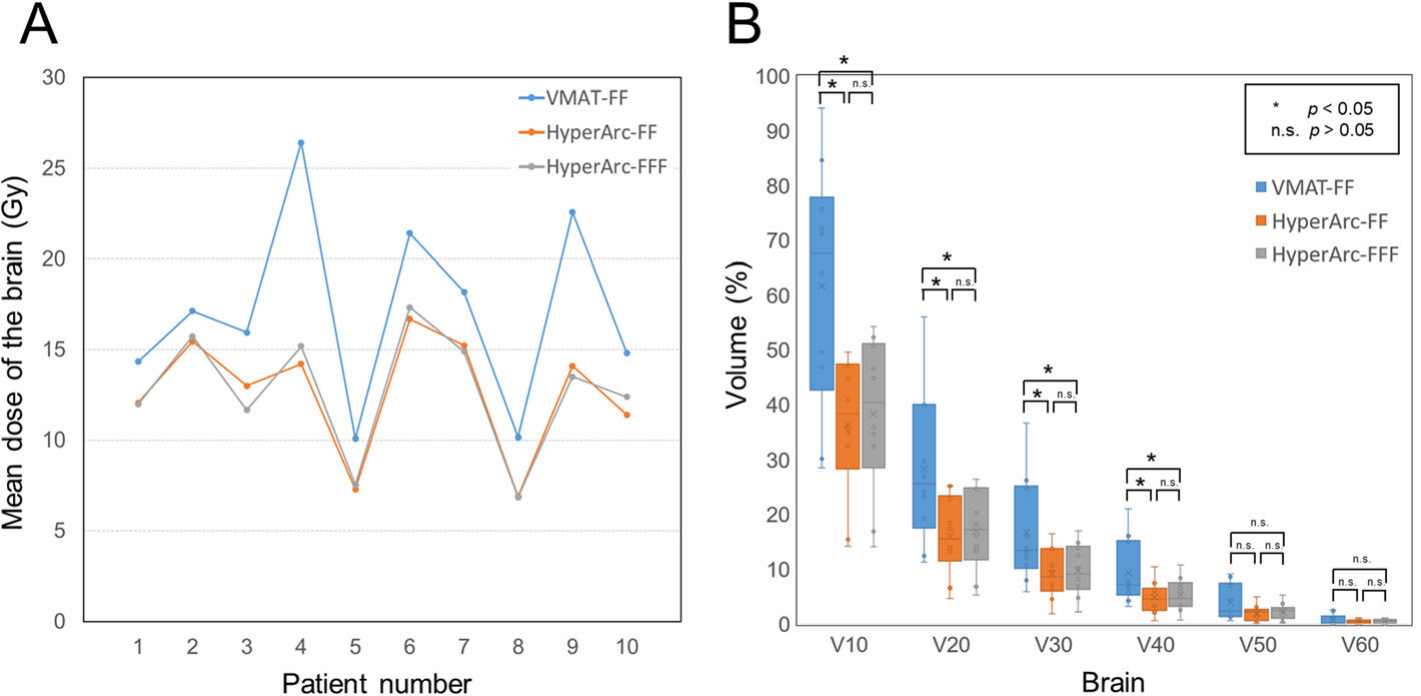
**a** Mean dose of the normal brain tissue for the VMAT-FF, HyperArc-FF, and HyperArc-FFF plans in each patient. **b** Boxplots of the volumes of the normal brain tissue that received a specific dose in a range of 10 to 60 Gy for the VMAT-FF, HyperArc-FF, and HyperArc-FFF plans

Table 3 illustrates the dosimetric parameters of PTV and OARs for three different plans. There were no statistically significant differences among the three different plans in PTV coverage, CI, and HI. However, the mean dose received by the brain in the HyperArc-FF and HyperArc-FFF plans was significantly lower than that in the VMAT-FF plan. The dose receiving 0.1 cc of the volume for surrounding the brain stem in the HyperArc-FF and HyperArc-FFF plans was lower than that in the VMAT-FF plan. For the other OARs, such as the chiasm, optic nerve, and lens, no statistically significant differences are observed between the VMAT and HyperArc plans. In addition, there were no statistically significant differences between the HyperArc-FF and HyperArc-FFF plans in the dose received by OARs. Figure 3a shows the mean dose received by the brain tissue of all patients in the VMAT-FF, HyperArc-FF, and HyperArc-FFF plans. In all patients, the mean dose received by the brain tissue in the HyperArc plans was lower than that in the VMAT plan. The distribution of the brain dose is displayed in Fig. 3b. The HyperArc-FF and HyperArc-FFF plans provided significantly lower V_10Gy_ (36.30% ± 12.76 and 38.28% ± 14.22%), V_20Gy_ (16.01% ± 7.17 and 17.04% ± 7.42%), V_30Gy_ (9.09% ± 4.60 and 9.73% ± 4.74%), and V_40Gy_ (4.73% ± 2.88 and 5.22% ± 3.01%) than did the VMAT-FF plan (61.70% ± 22.22, 28.31% ± 16.07, 16.70% ± 9.58, and 9.27% ± 5.93% for V_10Gy_, V_20Gy_, V_30Gy_, and V_40Gy_, respectively).

**Table 3:** The mean values of dosimetric parameters of PTV and OARs for VMAT-FF, HyperArc-FF, and HyperArc-FFF plans

Structure	Dosimetric parameters	VMAT-FF	HyperArc-FF	HyperArc-FFF	***P*** value*
PTV	D_1%_ (Gy)	73.81 ± 1.53	73.98 ± 0.86	74.45 ± 1.07	a = n.s., b = n.s.
	D_2%_ (Gy)	73.44 ± 1.31	73.55 ± 0.85	74.12 ± 1.07	a = n.s., b = n.s.
	D_98%_ (Gy)	64.99 ± 1.56	65.09 ± 1.24	64.90 ± 1.18	a = n.s., b = n.s.
	D_99%_ (Gy)	64.07 ± 1.83	64.32 ± 1.15	63.91 ± 1.23	a = n.s., b = n.s.
	CI	0.44 ± 0.05	0.43 ± 0.06	0.42 ± 0.05	a = n.s., b = n.s.
	HI	0.12 ± 0.04	0.12 ± 0.02	0.13 ± 0.03	a = n.s., b = n.s.
Brain	D_mean_ (Gy)	17.11 ± 5.25	12.63 ± 3.31	12.71 ± 3.40	a = 0.005, b = n.s.
Brain stem	D_0.1cc_ (Gy)	9.28 ± 2.01	7.32 ± 1.05	7.87 ± 1.27	a = 0.028, b = n.s.
Chiasm	D_0.1cc_ (Gy)	7.67 ± 2.30	6.37 ± 1.08	6.71 ± 1.33	a = n.s., b = n.s.
Optic nerve Left	D_0.1cc_ (Gy)	9.56 ± 4.83	8.38 ± 3.82	8.47 ± 4.64	a = n.s., b = n.s.
Optic nerve Right	D_0.1cc_ (Gy)	9.36 ± 4.58	8.19 ± 4.56	7.12 ± 3.28	a = n.s., b = n.s.
Lens Left	D_0.1cc_ (Gy)	4.72 ± 1.59	4.25 ± 1.57	4.14 ± 1.46	a = n.s., b = n.s.
Lens Right	D_0.1cc_ (Gy)	4.94 ± 2.02	4.27 ± 1.59	4.25 ± 1.29	a = n.s., b = n.s.

*Abbreviation*: *n.s.* not significant

*P value corresponds to the paired Wilcoxon signed-rank test: a = VMAT-FF vs HyperArc-FF, b = HyperArc-FF vs HyperArc-FFF

Table 4 summarizes the mean values of MCSV, total MUs, and BOT for VMAT-FF, HyperArc-FF, and HyperArc-FFF plans. The HyperArc-FF and HyperArc-FFF plans provided significantly lower the MCSV than did the VMAT-FF plan. The HyperArc-FFF plan had the highest total MUs among three different plans. The HyperArc-FF and HyperArc-FFF plans provided shorter BOTs than did the VMAT-FF plan. Moreover, the dosimetric verification using the EPID and gamma method with the PerFRACTION software was performed in all plans in this study. The mean gamma pass rates with 3%/2 mm and 2%/2 mm criteria in the VMAT-FF, HyperArc-FF, and HyperArc-FFF plans were 99.98% ± 0.03 and 99.91% ± 0.11, 99.91% ± 0.17 and 99.44% ± 0.82, and 99.84% ± 0.27 and 99.41% ± 0.86%, respectively.

**Table 4:** The mean values of MCSV, MU, and BOT for VMAT-FF, HyperArc-FF, and HyperArc-FFF plans

Parameters	VMAT-FF	HyperArc-FF	HyperArc-FFF	***P*** value*
MCSV	0.31 ± 0.02	0.27 ± 0.02	0.27 ± 0.02	a = 0.017, b = n.s.
Total MUs	863 ± 191	1025 ± 135	1206 ± 195	a = 0.041, b = 0.007
BOT (s)	380 ± 79	224 ± 29	217 ± 32	a = 0.005, b = n.s.

*Abbreviation*: *n.s.* not significant

*P value corresponds to the paired Wilcoxon signed-rank test: a = VMAT-FF vs HyperArc-FF, b = HyperArc-FF vs HyperArc-FFF

## Discussion

Previous studies have shown the superiority of HyperArc planning with regard to generating high conformity and rapid dose fall-off in only SRS [14, 16–18]. This is the first study to evaluate the performance of HyperArc planning technique in TSI for angiosarcoma. We designed three different non-coplanar plans (VMAT-FF, HyperArc-FF, and HyperArc-FFF) and evaluated the dose distribution, dosimetric parameters, and dosimetric accuracy for angiosarcoma of the scalp.

Firstly, we compared the dose distribution and dosimetric parameters between VMAT-FF and HyperArc-FF plans. It was evident that the dose distribution in the HyperArc-FF was superior to that in the VMAT-FF. Moreover, the mean dose of the brain in the HyperArc-FF plan for each patient was significantly lower than that in VMAT-FF plan. This is because the role of the SRS NTO in HyperArc plan was so powerful in producing the steep dose gradient in the distance perpendicular to the surface of the target. In addition, Hu et al. found that the mean dose of the brain in non-coplanar VMAT plan for TSI was about 19.2 Gy [9], and a similar result was observed with regard to VMAT-FF in this study. These results indicate that the HyperArc plan can reduce the dose received by normal brain tissue compared to the VMAT plans.

Secondly, to assess the utility of the FFF beams, we compared the dosimetric parameters between the HyperArc-FF and HyperArc-FFF plans. FFF beams have much higher dose rate and lower peripheral dose than do FF beams, which resulted in the reduction of out of field dose occurring because of head scatter and electron contamination [19]. Liu et al. found that coplanar VMAT with FFF beams reduced the dose received by OARs more than coplanar VMAT with FF beams in TSI [20]. In this study, however, it is interesting to note that the HyperArc-FFF and HyperArc-FF plans showed almost equal OAR sparing. This may be because common non-coplanar beams show a better conformity of the target and higher reduction of the dose received by OARs than do coplanar beams; furthermore, non-coplanar beams have a stronger effect than do FFF beams, that is, effects of the former may have masked those of the latter. These results indicated that there is almost no difference in the OAR sparing between the HyperArc planning using either FF or FFF beams.

In this study, the HyperArc-FF and HyperArc-FFF plans showed significantly lower MCSV compared to the VMAT-FF plan. The HyperArc plan increased the complexity of the MLC, but there was no effect on the dose delivery since the gamma test pass rates were above 95% in all plans. Therefore, introducing the HyperArc plan into clinical practice for TSI should not pose any problems.

The BOT in the VMAT-FF plan was about 1.5 times longer than that in the HyperArc-FF and HyperArc-FFF plans. This is because the number of arcs in the VMAT-FF plan was more than that in the HyperArc-FF and HyperArc-FFF plans. Moreover, HyperArc can reduce the overall treatment time because of the automated couch rotation movement during the treatment. Therefore, it is suggested that the HyperArc planning can be used to reduce the risk of patients’ intrafractional setup errors, such as the patient movement, compared with the non-coplanar VMAT planning. However, there was little difference in the utility between the HyperArc-FFF and HyperArc-FF plans in BOT. The potential for a higher dose rate of FFF beams could not be shown because BOT was governed mostly by the gantry speed. In addition, total MUs in the HyperArc-FFF plan was about 1.4 and 1.2 times higher than that in the VMAT-FF and the HyperArc-FF plans, which was caused by the special non-flat profile of the FFF beams. Accordingly, there is little necessity to choose the FFF beams in the HyperArc planning for TSI.

Previous studies reported that a total dose of 70 Gy in 35 fractions was typically delivered to patients with angiosarcoma of the scalp that is highly malignant and associated with a very poor prognosis owing to distant metastases at an early stage [1–5]. Accordingly, it is important to reduce the radiation damage to the normal brain tissue occurring because of a higher prescription dose in such cases than in other TSI cases, such as cutaneous lymphoma and squamous cell carcinoma. In addition, several studies have reported that the retention of the neurocognitive function is associated with the reception of a low dose by the brain and the radiation damage to the hippocampus plays a considerable role in neurocognitive function decline [21, 22]. In this study, we could not delineate the hippocampus because we did not get the magnetic resonance imaging (MRI) imaging for the OARs contouring in the process of simulation. However, we proved the potential of the HyperArc planning in reducing the low dose received by the brain, which results in protection of the neurocognitive function. Using the contour of the hippocampus in the HyperArc planning could lead to a better sparing of the hippocampus. Furthermore, Song et al. reported that the HT was a better modality for TSI than the coplanar VMAT because it provided better target coverage and conformity with lower brain doses [10]. However, this report was performed in only one patient and the result of the BOT in HT plan was over 10 min, indicating that the BOT was over 2.5 times longer than that in the HyperArc plan in our study. Further investigation should be carried out in order to determine which modality is best for TSI.

Several limitations of this study should be mentioned. Angiosarcoma of the scalp is a rare malignancy, and the patients who underwent TSI were very few cases. Various reports have included a small number of patients who have undergone TSI because of the same reason. Therefore, the small sample size and the variation of dosimetric parameters between patients in this study need to be considered while interpreting the results. In addition, non-coplanar beam in the HyperArc planning was determined from the given four couch rotation angles. An increase in the couch angle may improve the quality of the HyperArc planning for TSI. Despite these limitations, our quantitative data makes an important contribution to the field of radiation therapy with regard to TSI.

## Conclusion

This study clearly demonstrates the superiority of the HyperArc with regard to dose distribution, OAR sparing while maintaining the target coverage, and BOT, in comparison with the non-coplanar VMAT for TSI in angiosarcoma of the scalp. There were no advantages of the dosimetric parameters in the HyperArc planning with FFF beams compared to that with FF beams. Our study suggests that the HyperArc plan has the potential to be employed for radiation therapy when target regions with large and complicated shapes such as the scalp, are involved.

## Data Availability

The datasets used and/or analyzed during the current study are available from the corresponding author on reasonable request.

## Abbreviations

AAA: Analytical anisotropic algorithm
AAV: Aperture area variability
BOT: Beam-on time
CI: Conformity index
CT: Computed tomography
CTV: Clinical target volume
EPID: Electronic portal imaging device
FF: Flattening filter
FFF: Flattening filter free
HI: Homogeneity index
HT: Helical tomotherapy
LSV: Leaf sequence variability
MCSV: Modulation complexity score for volumetric modulated arc therapy
MLCs: Multileaf collimeters
MRI: Magnetic resonance imaging
MU: Monitor Unit
NTO: Normal tissue objective
OAR: Organ at risk
PO: Photon optimizer
PTV: Planning target volume
PV: Prescription isodose volume
SRS: Stereotactic radiosurgery
TPS: Treatment planning system
TSI: Total scalp irradiation
TV: Target volume
TV_PV_: Volume of the target covered by the prescription dose
VMAT: Volumetric modulated arc therapy
